# Spinal Cord Compression Revealing an Intraosseous Schwannoma

**DOI:** 10.1155/2013/913218

**Published:** 2013-12-05

**Authors:** Leila Metoui, Faïda Ajili, Mouna Maiza, Mehdi Ben Ammar, Imen Gharsallah, Issam M'sakni, Bassem Louzir, Salah Othmani

**Affiliations:** ^1^Department of Internal Medicine Military Hospital, Montfleury, 1008 Tunis, Tunisia; ^2^Department of Neurosurgery Military Hospital, Montfleury, 1008 Tunis, Tunisia; ^3^Department of Anatomopathology Military Hospital, Montfleury, 1008 Tunis, Tunisia

## Abstract

A 68-year-old female presented with inflammatory lumbalgia and cruralgia. Physical examination revealed a lumbar stiffness without neurological deficit. Secondarily, paraplegia and urinary retention appeared. Magnetic resonance imaging showed a vertebral compaction of L3 vertebra with medullar compression. Emergent surgery revealed an epidural tumor involving largely the L3 vertebral body. Histology found schwannoma with positive protein S100 on the immunohistochemical study. Metastasis screening revealed bilateral nodular lesions of the lungs and a trochanter high scintigraphic signal. It was a malignant schwannoma. The patient underwent radiotherapy in addition to the total tumor resection.

## 1. Introduction

Schwannomas are nerve tumors that might be benign or transformed to a malignant peripheral nerve sheath tumor (MPNST). Schwannomas present almost third of primary spinal tumors. Intraosseous localization is rare, reported as case reports. We report a new case of malignant spinal intraosseous schwannoma.

## 2. Case Report

**Figure 1:**
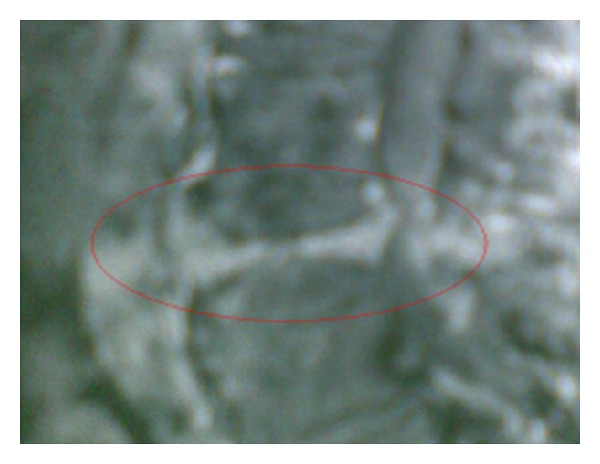
MRI: T1 low sign: L3 compaction.

**Figure 2:**
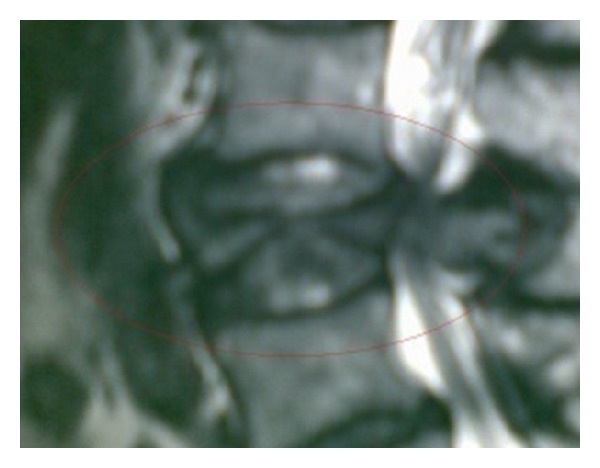
T2 high sign with dural sac compression.

**Figure 3:**
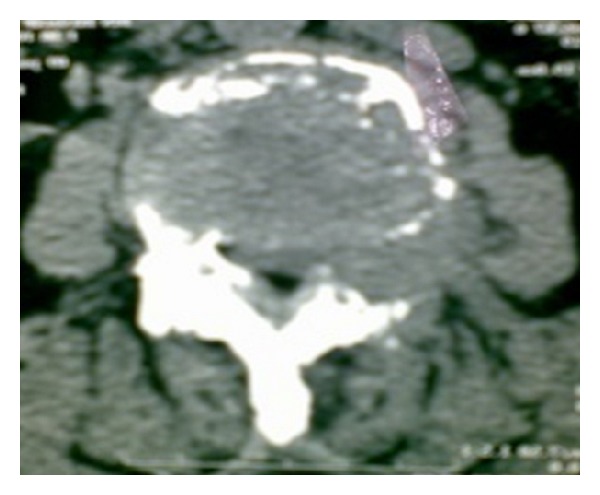
L3 compaction.

**Figure 4:**
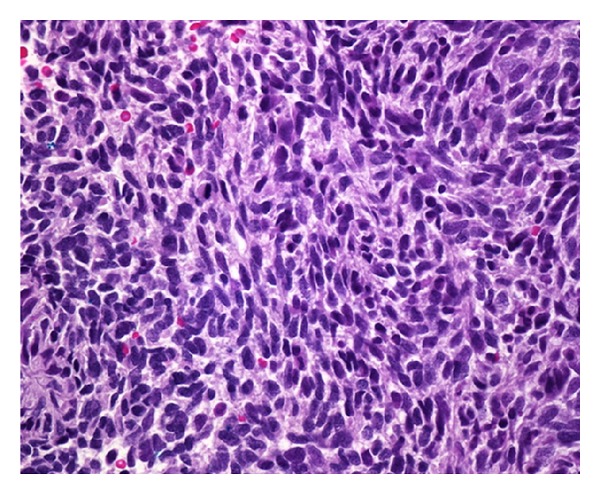
Spindle-shaped cells.

**Figure 5:**
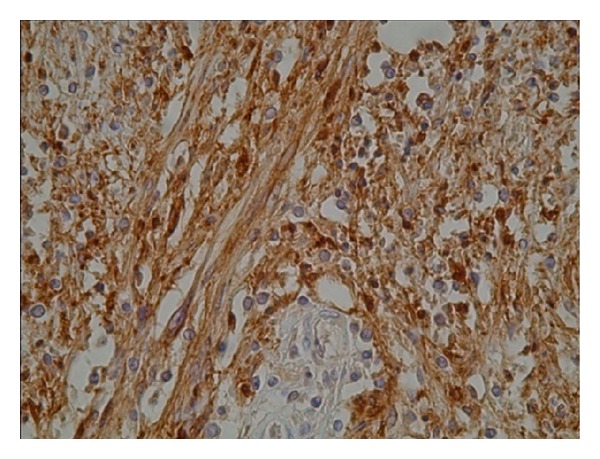
Protein S100 positivity on the immunohistochemical study.

A 68-year-old female, with no previous medical problems, presented with inflammatory lumbalgia and cruralgia which are progressively increasing since three months. She had no genital or sphincter dysfunction. Physical examination revealed a lumbar stiffness without neurological deficit. During hospitalization, paraplegia and urinary retention appeared. Magnetic resonance imaging showed a vertebral compaction of L3 vertebra with decline of the posterior vertebral wall and compression of the dural sac (Figures 1, 2, and 3). A surgical medullar decompression was indicated emergently. An epidural tumor involving largely the L3 vertebral body was found and successfully resected. Histological findings were suggestive of schwannoma because of the presence of spindle-shaped cells (Figure 4). Protein S100 positivity on the immunohistochemical study confirmed the diagnosis of schwannoma (Figure 5). The patient underwent a thoracoabdominal tomodensitometry which revealed bilateral nodular lesions of the lungs. A total bone scintigraphy found high signal of the L3 vertebra and the trochanter. Pulmonary nodules were considered metastatic and not the primitive tumor because they were multiple and infracentimetric; getting biopsy of these pulmonary nodules would be important to confirm this, but it was impossible because of the size. This biopsy would also be able to show if these nodules corresponded to schwannomas which defines a probable schwannomatosis. This hypothesis cannot be definitely ruled out despite the absence of a first-degree relative with schwannomatosis and the absence of cranial nerve involvement. So, our patient was considered to have a malignant intraosseous schwannoma with probable lung metastases. Consequently, radiotherapy was associated.

## 3. Discussion

Spinal schwannomas account for 30% of primitive spinal tumors [1]. They are usually intradural (70%) and less frequently extradural [2, 3]. The intraosseous localization is rare. Schwannomas present less than 0,2% of primitive bone tumors [3]. Bones contain a few of myelinated nerve fibers. This may explain the rarity of this tumor [4, 5]. Some mechanisms of bone involvement have been postulated: a bone erosion caused by an extraosseous tumor, a centrally arising tumor, and a nutrient canal arising tumor causing its enlargement [6, 7]. The mandible is the most prevalent involved bone [4, 6, 8]. Other localizations were described including long bones, cranial bones, ribs, scapula, sacrum, calcaneum, and small bones of the hands [7, 9–13]. Vertebral localization could be explained by developmental entrapment of some of the neural crest cells into the vertebral body, which differentiate along the Schwann cell lineage and later transformation leads to the MPNST [14]. Spinals chwannoma was first reported in 1964 [3]. The lumbar site is more frequent (44%) than the cervical site (31%) and the thoracic site (25%) [3]. Spinal schwannoma develops usually from the vertebral body. Nevertheless, a case of a posterior arc schwannoma was reported [6]. Intraosseous spinal schwanomas sizes are usually larger than nonintraosseous ones and their borders are irregular suggesting an invasive potential of these tumors [3]. Their progression induces pedicel and corporeal erosion with enlargement of the nerve root foramina, but important corporeal destruction remains rare like in our case [7]. Medullar compression is possible. Schwannomas are typically well-limited, encapsulated, and lobulated tumors [7]. Histologically, there are two types of tissues: Antoni type A tissue consisting of compact cellular area of spindle-shaped cells arranged in bundles or fascicles and Antoni type B tissue which is less cellular with hazardly arranged spindle-shaped cells [15]. In immunohistochemical study, protein S100 is positive [16, 17]. Clinical and radiological features cannot predict the malignancy or the benignity of the tumor [2].

Schwannomas seem to appear between the third and the sixth decades [16]. The mean age of osseous schwannomas is 41,1 years [3]. They are slightly more common in males [3]. Association with neurofibromatosis is possible in 10% of cases and may be related to the type II neurofibromatosis gene (NF2) [16]. Presence of multiple schwannomas in the same patient is possible during schwannomatosis which is a predisposing condition to malignant peripheral nerve sheath tumors; in our case, there was no evidence of schwannomatosis and there was no medical history of familiar schwannomatosis. But the pulmonary nodules could be schwannomas, unfortunately, pulmonary biopsy was not possible and our patient might have schwannomatosis. Pulmonary nodules could also be metastatic from the intraosseous transformed schwannoma. A similar case was reported in a 28-year-old man, without any stigmata of von Recklinghausen's disease, presenting a femoral spindle cells tumor with a nonbiopsied pulmonary nodule [18]. Multiple cervical primary intraosseous MPNSTs were reported in a 41-year-old male presented with a 1-month history of radiating pain to his right shoulder and arm [19]. An intraosseous MPNST of the thoracic spine causing cord compression was reported in a 59 year-old woman presented with midthoracic back pain and a T4 sensory level. MRI showed a destructive lesion at the T3 level with cord compression, decompressive laminectomies, tumor debulking and instrumentation were performed [20]. Another thoracic MPNST was described in a 75-year-old male; the MPNST was arising from the body of a thoracic spine with a minimal intraspinal component and associated with a huge tumor part occupying the paraspinal and retrospinal region [14].

Symptoms of spinal schwannoma are various including lumbar and root pain, motor deficit of variable degree, and genitosphincter dysfunction. Magnetic resonance is an excellent imaging exam in schwannomas. It usually finds high signs on T2 weighted images and shows possible osseous destructions and extraosseous progression [1, 13]. Intraosseous schwannoma treatment is based on surgical excision. The surgical technique depends on the size and the location of the tumor and may cause spinal instability that can be avoided with the use of adequate instruments [2, 3]. Neurological recuperation is possible after surgery [21]. Spinal tumors surgery can also be complicated with local pain or neurological deficit [22]. Malignant schwannoma outcome is low with short survival [2]. Recurrence of malignant schwannoma is possible even after total removing of the tumor [2]. Malignant schwannomas are usually resistant to chemotherapy and radiotherapy (in [23]).

## 4. Conclusion

Intraosseous schwannoma is a rare curable nerve tumor. Magnetic resonance is the best indicated imaging. The diagnosis confirmation is histological and immunohistochemical. Other localizations or familiar cases must be searched in order to diagnosis schwannomatosis. Total surgical removal is the treatment. The outcome is good in benign forms but malignant forms have low outcome despite surgery and chemotherapy or radiotherapy.
